# Advances in Diagnosing and Managing Primary Systemic Vasculitides: A Transforming Landscape

**DOI:** 10.3390/diagnostics16050788

**Published:** 2026-03-06

**Authors:** Alicia Rodriguez-Pla

**Affiliations:** Premier Rheumatology, P. C., 1189 E Herndon Avenue, Suite 101, Fresno, CA 93720, USA; arp@premierrheumatology.org

As we conclude this Special Issue of *Diagnostics*, “Advances in the Diagnosis and Management of Vasculitis,” we reflect on a vibrant collection of eleven articles that span the globe from Spain and Poland to the USA, Germany, Romania, and Turkey. This international collaboration underscores a universal knowledge in rheumatology: while vasculitis remains a diagnostic “chameleon,” our collective ability to understand it has never been stronger [1,2].

The primary systemic vasculitides occupy a unique space in medicine—rare enough to challenge timely recognition, and serious enough to demand urgent, evidence-based action. Despite the significant progress over the past two decades, the path from symptom onset to accurate diagnosis remains fraught with complexity, and therapeutic strategies continue to evolve at an unprecedented pace. The approval of avacopan for anti-neutrophil cytoplasmic antibody (ANCA)-associated vasculitis (AAV) in 2021 [3], the Food and Drug Administration (FDA) approval of upadacitinib for giant cell arteritis (GCA) in April 2025 [4], and the accelerating integration of advanced imaging modalities into clinical workflows have collectively reshaped how we conceptualize and manage these diseases [5]. This Special Issue of *Diagnostics* was conceived to capture this momentum—bringing together original research, reviews, and case reports that address diagnostic challenges, emerging biomarkers, novel imaging approaches, treatment strategies, and patient outcomes across the vasculitis spectrum.

## 1. Diagnostic Complexity of Giant Cell Arteritis

GCA remains the most common systemic vasculitis in adults over 50 years of age, yet its heterogeneous presentation continues to pose significant diagnostic hurdles [6]. Updated classification criteria, advances in imaging technologies, the evolving biomarker landscape, and emerging therapeutic strategies including the role of targeted therapies are rapidly transforming the management paradigm [7,8,9]. Importantly, no single pathognomonic feature defines GCA and that a multimodal approach integrating clinical assessment, laboratory evaluation, and imaging is essential to reduce the risk of complications such as irreversible vision loss [10].

The urgency of rapid diagnosis is illustrated by Stańska et al., who present a case report of a 74-year-old patient in whom diagnostic delays led to bilateral vision loss. Their work supports ultrasound as the first-line imaging tool for suspected GCA, in alignment with the 2023 European League Against Rheumatism (EULAR) recommendations that position color duplex sonography (PCDS) above temporal artery biopsy (TAB) for its sensitivity, non-invasiveness, and wide availability, while acknowledging that operator expertise remains a key limiting factor, and TAB remains a valuable tool in many clinical situations [11].

## 2. The Expanding Role of Imaging

Imaging has emerged as a central pillar in vasculitis diagnosis and monitoring. The role of positron emission tomography/computed tomography (PET/CT) in GCA has been established, especially in specific clinical scenarios—including atypical presentations, suspicion of large vessel involvement, and diagnostic uncertainty in the setting of mimickers—in which this imaging modality can provide decisive information [12]. Color duplex ultrasound and magnetic resonance imaging have also been found to be helpful tools for diagnosing GCA [13].

## 3. Rare Presentations and Diagnostic Mimickers

The vasculitis spectrum extends beyond GCA and AAV. In the arena of monogenic vasculopathies, [14] contributed an important case report on hepatic vascular involvement in deficiency of adenosine deaminase 2 (DADA2), describing large regenerative nodules with a focal nodular hyperplasia (FNH)-like appearance not previously reported in this setting. Notably, etanercept therapy resulted in sustained clinical stabilization and the marked regression of liver lesions over nine years of follow-up, expanding our understanding of the hepatic manifestations and treatment responsiveness in this rare condition.

## 4. ANCA-Associated Vasculitis: From Pathogenesis to Prognosis

Significant advances in biomarker research in AAV is helping to change the treatment paradigm [15,16]. In the context of the expanding therapeutic armamentarium—including avacopan, which was found to be superior to prednisone in sustained remission at 52 weeks in the ADVOCATE trial —the ability to stratify patients by relapse risk has direct implications for treatment selection and monitoring intensity [17].

## 5. Nephritis in IgA Vasculitis: Outcomes Under Immunosuppression

Yıldırım et al. contributed a prospective observational study of 49 adults with IgA vasculitis nephritis (IgAVN), an area with a notable absence of specific management guidelines. Their findings challenge the prevailing perception of uniformly poor renal outcomes in adults, demonstrating that ESRD occurred in only one patient (2%) when effective immunosuppressive therapy was employed. The identification of nephrotic-range proteinuria as an independent risk factor for unfavorable outcomes (OR: 17.18) provides a practical prognostic marker. Importantly, the study also highlights the infection risk associated with immunosuppression, particularly in older adult patients—a reminder that therapeutic gains must be weighed against treatment-related morbidity [18].

## 6. A Transformative Era

Collectively, these 11 contributions illustrate that the field of vasculitis is undergoing a transformation driven by converging advances in imaging, immunopathogenesis, targeted therapeutics, and clinical phenotyping. The expanding application of US, PET/CT, and contrast-enhanced ultrasound (CEUS) across the vasculitis spectrum enables earlier diagnosis, more precise disease monitoring, and better-informed therapeutic decision-making [19,20]. The common thread among these diverse papers, ranging from prospective studies in Turkey to retrospective cohorts in Spain, is the urgent need to reduce treatment toxicity without compromising efficacy. The approval of complement inhibitors such as avacopan for AAV [21] and JAK inhibitors such as upadacitinib for GCA [4] mark a paradigm shift toward mechanism-based, steroid-sparing strategies to offer a roadmap for improving long-term outcomes and quality of life.

However, many unresolved questions persist. The optimal duration and intensity of immunosuppression, the role of imaging in treatment de-escalation, the identification of validated biomarkers for disease activity and relapse prediction, and the development of tailored approaches for rare vasculitis phenotypes all require continued investigation. The diversity of contributions in this Special Issue—spanning LVV, small-vessel vasculitis, IgA vasculitis, IgG4-RD, and monogenic vasculopathy—reflects the breadth of the challenges and the vitality of the research community dedicated to addressing them.

I am deeply grateful to all contributing authors for their rigorous work and to the reviewers whose constructive assessments strengthened each manuscript. I hope that this collection serves as both a practical resource for clinicians and a catalyst for future study, advancing us closer to the ultimate goals: timely diagnosis, personalized treatment, and improved outcomes and quality of life for all patients with vasculitis.
